# The Emerging Role of DNA Damage in the Pathogenesis of the C9orf72 Repeat Expansion in Amyotrophic Lateral Sclerosis

**DOI:** 10.3390/ijms19103137

**Published:** 2018-10-12

**Authors:** Anna Konopka, Julie D Atkin

**Affiliations:** 1 Centre for MND Research, Department of Biomedical Sciences, Faculty of Medicine & Health Sciences, Macquarie University, Sydney, NSW 2109, Australia; anna.konopka@mq.edu.au; 2 La Trobe Institute for Molecular Science, Melbourne, VIC 3086, Australia

**Keywords:** C9orf72, ALS, motor neuron disease, R loops, nucleolar stress, neurodegeneration

## Abstract

Amyotrophic lateral sclerosis (ALS) is a fatal, rapidly progressing neurodegenerative disease affecting motor neurons, and frontotemporal dementia (FTD) is a behavioural disorder resulting in early-onset dementia. Hexanucleotide (G4C2) repeat expansions in the gene encoding chromosome 9 open reading frame 72 (*C9orf72*) are the major cause of familial forms of both ALS (~40%) and FTD (~20%) worldwide. The C9orf72 repeat expansion is known to form abnormal nuclei acid structures, such as hairpins, G-quadruplexes, and R-loops, which are increasingly associated with human diseases involving microsatellite repeats. These configurations form during normal cellular processes, but if they persist they also damage DNA, and hence are a serious threat to genome integrity. It is unclear how the repeat expansion in *C9orf72* causes ALS, but recent evidence implicates DNA damage in neurodegeneration. This may arise from abnormal nucleic acid structures, the greatly expanded C9orf72 RNA, or by repeat-associated non-ATG (RAN) translation, which generates toxic dipeptide repeat proteins. In this review, we detail recent advances implicating DNA damage in C9orf72-ALS. Furthermore, we also discuss increasing evidence that targeting these aberrant *C9orf72* confirmations may have therapeutic value for ALS, thus revealing new avenues for drug discovery for this disorder.

## 1. Introduction

Maintaining the stability and integrity of the genome is essential for normal cellular viability. Damage to DNA can arise from both endogenous and exogenous sources, and every cell receives numerous DNA injuries per day [1]. These injuries can generate mutations and compromise cellular viability, so safeguarding genetic integrity is of fundamental importance to human health [1]. DNA damage occurs in many forms. Single-stranded breaks (SSBs) involve a cut in the phosphodiester backbone of one DNA strand, whereas both DNA strands are severed in double-stranded breaks (DSBs). Although DSBs are much less common than SSBs, DSBs are difficult to repair and are the most cytotoxic lesion [2]. Alternatively, mismatch or modification of individual bases is another form of DNA damage [2].

Neurons are particularly vulnerable to DNA damage, because they are post-mitotic with high metabolic rates, and they are highly susceptible to oxidative stress, which is a major source of DNA damage [3]. Furthermore, SSBs are predicted to be more detrimental in post-mitotic neurons than in other cell types, because there are fewer options for repairing SSBs compared to proliferating cells. Hence, SSBs in neurons are more likely to be converted to highly cytotoxic DSBs than in other cell types [4]. In addition, DNA damage increases with advancing age, which is a major risk factor for neurodegenerative disorders [5].

DNA damage is now well-documented in neurodegenerative diseases, including ataxia-telangiectasia, Parkinson’s disease, and Alzheimer’s disease [6,7]. Dysfunctional DNA repair and DNA damage is also a growing area of interest in amyotrophic lateral sclerosis (ALS). The greatest proportion of familial cases of ALS are caused by a hexanucleotide repeat expansion in the gene encoding chromosome 9 open reading frame 72 (*C9orf72*). This review will focus on recent findings revealing a relationship between the formation of abnormal DNA structures, DNA damage, nucleolar stress, and C9orf72-ALS. These studies highlight the importance of genomic integrity in maintaining neuronal viability and they demonstrate a role for DNA damage in the pathogenesis of ALS.

## 2. Amyotrophic Lateral Sclerosis

ALS is a rapidly progressing and ultimately fatal neurodegenerative disorder affecting both upper motor neurons in the motor cortex and lower motor neurons in the brainstem and spinal cord. [8]. The clinical symptoms are varied, but involve progressive muscle weakness, spasticity, fasciculations, and eventually extensive paralysis [9], resulting in death from respiratory muscle failure usually within 2–5 years of diagnosis [10]. ALS is closely related to frontotemporal dementia (FTD), which affects the frontal lobes of the brain, and is characterised by behavioural changes in personality, emotion, and behaviour. FTD is diagnosed in approximately 20% of ALS cases [11], and an overlap between ALS and FTD exists at the clinical, genetic, and pathological levels. In fact, the discovery of the *C9orf72* mutation in both ALS and FTD confirmed that these two disorders represent opposite extremes of the same, continuous clinical disease spectrum [12]. Approximately 10% of ALS cases are caused by dominantly inherited mutations (familial ALS), unlike most cases, which arise sporadically (sALS, 90% of cases). The aetiology of ALS/FTD remains unclear, and the disease is thought to involve both environmental and genetic components.

Hexanucleotide repeat expansions (GGGGCC) in a non-coding region of *C9orf72* are the most common genetic abnormality in both ALS and FTD, which is responsible for approximately 40% of familial ALS, 5–10% of sporadic ALS, 40% of familial FTD, and 4–21% of sporadic FTD [13,14,15]. Mutations in the genes encoding the TAR-DNA binding protein (TDP-43) (*TARDBP*), fused in sarcoma (FUS) (*FUS*), TANK-binding kinase-1 (*TBK-1*), Ubiquilin 2 (*UBQLN2*), optineurin (*OPTN*), and Cyclin F (*CCNF*), are also present in both ALS and FTD patients [16,17,18,19,20,21,22,23,24,25,26,27,28,29,30,31]. In contrast, mutations in other genes are present in ALS only, including *SOD1* and *VAPB,* encoding superoxide dismutase 1 and VAMP (vesicle-associated membrane protein)-associated protein B and C, respectively [32]. 

As in other neurodegenerative diseases, the pathological hallmark of ALS is the presence of misfolded protein inclusions in affected tissues [33]. In ALS patients, motor neurons contain these inclusions, and in familial forms of disease, the inclusions contain the specific proteins that are mutated in each case [34]. In sporadic ALS, these inclusions contain several different proteins, including wildtype, misfolded, TDP-43, which is also ubiquitinated, hyper-phosphorylated, and aberrantly mis-localised from the nucleus to the cytoplasm [35]. In fact, this pathological form of TDP-43 is present in motor neurons of almost all cases of ALS/FTD (97%) [36]. Similarly, TDP-43 pathology is present in 45% of FTD cases, implying that TDP-43 is a signature pathological lesion in both ALS and FTD [37]. TDP-43 is also strikingly similar to FUS in terms of its normal cellular functions and its pathological characteristics. Both TDP-43 and FUS are heterogenous nuclear riboproteins (hnRNPs) that perform multiple roles in RNA processing, including alternative splicing and regulation of transcription and translation [38]. The ALS mutations in FUS are primarily found in the nuclear localisation signal (NLS); like TDP-43, FUS mislocalises from the nucleus to the cytoplasm, where it forms stress granules and aggregates [39,40,41]. 

The accumulation of misfolded proteins in ALS implies that dysfunctional protein homeostasis (proteostasis) mechanisms are central to pathogenesis, and several of these processes are implicated in neurodegeneration, including defects in protein degradation (autophagy and the proteasome), protein trafficking (particularly nucleo-cytoplasmic transport), and protein folding. Similarly, several of the genes mutated in ALS encode proteins that function in proteostasis. However, the growing abundance of RNA binding proteins linked to ALS/FTD has also revealed a role for abnormal RNA metabolism in pathophysiology [42]. Whilst these two mechanisms are often highlighted as being central to ALS, this rather simplistic division does not fully capture the complexity of the range of functions performed by the proteins associated with ALS, nor the cellular signalling pathways that are known to be dysfunctional in this disorder. Recently, DNA damage has been linked to ALS [40,43,44,45,46,47]. Interestingly, many of the signalling pathways associated with DNA damage are also implicated in ALS, including oxidative stress, mitochondrial function, RNA metabolism, autophagy, and proteosomal function [48,49,50,51,52].

## 3. DNA Damage Signalling

Cells have developed elaborate signalling systems to detect and repair damage to DNA, termed the “DNA damage response” (DDR). Many normal physiological events induce DNA damage, particularly transcription and mitochondrial respiration, which generates reactive oxygen species [53]. It is therefore essential that the cell normally maintains genomic stability. Depending on the extent of damage and risk of mutation, the cell induces DNA repair pathways, or following chronic activation of DDR, induces apoptosis to protect the organism. Various sensor proteins detect DNA damage, and the most widely used sensor experimentally is the phosphorylated histone variant H2AX (γH2AX). The formation of SSBs or DSBs activates phosphorylation of H2AX, hence γH2AX flanks sites of DNA damage. This initiates recruitment of the ataxia-telangiectasia mutated (ATM) and Rad3-related (ATR) protein kinases, which trigger the DDR. Poly (ADP-ribose) polymerase (PARP) and p53 binding protein (53-BP1) are other important sensors that signal DNA damage during DDR. Depending on the type of DNA damage, the DDR can activate several different DNA repair pathways. SSBs are repaired primarily by excision repair mechanisms, whereas DSBs are repaired by either homologous recombination (HR) or non-homologous end-joining (NHEJ). However, in neurons, NHEJ is the primary mechanism, because HR requires active progression through the cell cycle [54,55]

The nucleolus is a prominent cellular compartment located within the nucleus, and it is implicated in the DDR because it contains over 160 DNA repair proteins [56]. However, it is unclear whether the nucleolus is simply a storage facility for DDR proteins, or if these proteins have specific roles in DNA repair in the nucleolus. The nucleolus is also responsible for the biogenesis of ribosomes and the regulation of cellular stress responses.

## 4. DNA Damage and Neurodegeneration

Nucleotide repeat elements are common in the eukaryotic genome. Microsatellite (short-repeat) expansions are responsible for almost 40 different diseases, including many neurological disorders [57,58]. These repeat expansions lead to instability of the repeat and the formation of abnormal DNA structures. Interestingly, mutations in DNA repair genes lead to disorders that produce neurological phenotypes: xeroderma pigmentosum and ataxia-telangiectasia [59]. Furthermore, defects in DNA repair can be manifested primarily in neural tissues, leading to neurological conditions [60]. It is therefore not surprising that DNA damage has also been detected in neurodegenerative disorders, including Alzheimer’s, Parkinson’s, and Huntington’s diseases [6,7], as well as ALS. These findings therefore imply a close relationship between DNA damage/repair and neuronal function.

Most previous historical studies on DNA damage in ALS were examined in relation to oxidative stress, which occurs in mitochondrial rather than nuclear DNA; they also preceded the discovery of the relationship between TDP-43, C9orf72, and ALS. DNA damage was present in sporadic ALS patients in regions of the CNS that contain motor neurons, but not in other regions [61]. Apoptosis in spinal motor neurons follows DNA damage [62] and DNA repair enzymes are up-regulated in the brain, indicating increased DNA damage [63]. Similarly, in ALS mouse models based on transgenic expression of mutant SOD1, motor neuron degeneration was associated with DNA damage [64], although these animals do not possess the TDP-43 pathology present in almost all ALS cases. DNA damage has also been detected in neuronal cells expressing G93A mutant SOD1 [65]. DNA repair activity detected by 8-hydroxy-2-deoxyguanosine (OHdG) immunoreactivity is also increased in the motor cortex of sALS patients [66], and elevated levels of 8-OHdG were also identified in familial ALS spinal cords bearing SOD1 mutations [66]. Apurinic/apyrimidinic endonuclease 1 (APE1) is elevated in the brain and spinal cord of sporadic ALS patients [45]. Activation of cellular DNA repair processes has been previously implicated in motor neuron degeneration [61,67], and mice lacking the gene encoding ERCC1, which is essential for SSB nucleotide excision repair and repair of DSBs, show age-related motor dysfunction [68].

Further evidence linking DNA damage to ALS is the increasing number of proteins mutated in this disorder that possess normal cellular functions in DNA repair, particularly FUS. DNA damage is present in transgenic mice expressing ALS-associated mutant FUS-R521C in cortical neurons and spinal motor neurons [69]. DSBs trigger FUS phosphorylation by ATM and DNA-dependent protein kinase (DNA-PK), both of which are involved in the DDR [70,71]. FUS binds to histone deacetylase 1 (HDAC1), and therefore indirectly regulates HR and NHEJ in primary mouse neurons [72]. ALS-associated FUS mutations in the nuclear localization sequence NLS cause impartment of PARP-dependent DDR, which leads to neurodegeneration and the formation of FUS aggregates [40]. FUS mutations are also responsible for defects in DNA nick ligation and oxidative damage repair in ALS patients [43]. Similarly, increased expression of γH2AX has been found in ALS patients carrying FUS-R521C or FUS-P525L mutations [72]. Moreover, FUS and TDP-43 function in the prevention or repair of transcription-associated DNA damage [73]. These findings therefore indicate that FUS is a DDR protein that functions in DNA repair, whereas in ALS, DNA repair is defective. However, despite the marked functional and pathological similarities between TDP-43 and FUS, a convincing role for TDP-43 in DNA repair has not yet been demonstrated.

Similarly, senataxin is a helicase that can resolve R-loops [74], and which is mutated in juvenile forms of ALS [75]. Mutations in other DNA damage/repair proteins are also present in more typical forms of ALS, such as valosin-containing protein (VCP) [76] and cyclin F [31]. VCP is implicated in the repair of DSBs [77], and cyclin F controls genome stability through ubiquitin-mediated proteolysis [78]. NIMA-related kinase 1 (NEK1), which is mutated in familial and sporadic ALS [22,79], is necessary for cellular checkpoint control in the DDR, independent of ATM or ATR [80], where it functions in replication fork stability in the completion of HR [81]. ALS-associated NEK1 mutations were shown to induce DNA damage in induced pluripotent stem cells (iPSC)-derived motor neurons [44]. C21ORF2 interacts with NEK1 and functions in HR, but not in NHEJ-mediated DSB repair, and it is also mutated in sporadic ALS [82]. 

## 5. Chromosome 9 Open Reading Frame 72 and Amyotrophic Lateral Sclerosis

Hexanucleotide repeat expansions in *C9orf72* are central to both ALS and FTD. Whilst the normal population bears fewer than eight GGGGCC repeats, and 50% of these individuals possesses only two repeats [83], in ALS and FTD this region is expanded up to several thousand times [84]. *C9orf72* uses alternative splicing to produce at least three different transcript variants. V2 and V3 encode a long isoform, whereas V1 encodes a short isoform. The repeat expansion is located either in intron 1 for transcripts V1 and V3 or within the promoter sequence for V2 [13]. Despite a common genetic cause, however, *C9orf72* repeat expansion carriers exhibit remarkably heterogeneous clinical and pathological characteristics. There also appears to be no unambiguous clinical correlation between the length of the repeat and disease onset or progression [85]. Interestingly, genetic analysis of the *C9orf72* repeat expansion has identified a common haplotype, but the lengths of the repeat vary among carriers. This implies that the repeats are either unstable and result from a single founder [86], or alternatively, that the repeat sequence is inherently prone to instability and results from different founders [87]. The *C9orf72* repeat expansion is linked to other neurological conditions including Alzheimer’s disease [88], multiple system atrophy [89], Huntington’s disease [90], cerebellar ataxia [91], multiple sclerosis [92], Parkinson’s disease [93], bipolar disorder [94,95], and schizophrenia [96].

The mechanism of how the *C9orf72* repeat expansion induces motor neuron death is unclear, but this may reflect the intronic nature of the repeat expansion. Three major mechanisms have been proposed, and their relative contributions to pathogenicity are consistently debated. Haploinsufficiency was initially proposed, given that *C9orf72* carriers express reduced levels of the *C9orf72* transcript compared to individuals without the repeat expansion [97]. However, mice with C9orf72 deficiency, or those expressing loss-of-function mutations, develop immune defects, increased expression of inflammatory cytokines, and autoimmunity [98,99], but no neurodegenerative phenotype, arguing against haploinsufficiency as a single causative factor in ALS/FTD. However, these findings may be reflective of the strong expression of C9orf72 in myeloid cells. Furthermore, a recent study concluded that both loss- and gain-of-function mechanisms cooperate and lead to neurodegeneration in ALS/FTD by a process involving the impairment of vesicle trafficking [100]. In contrast, there is more evidence in favour of gain of a toxic function in *C9orf72*-ALS/FTD. One possible mechanism is the induction of toxicity by transcription of the *C9orf72* repeat expansion, producing greatly expanded RNA, which forms predominately nuclear RNA foci in affected tissues [101]. These RNA foci are thought to sequester important RNA-binding proteins (RBPs), such as those involved in alternative splicing, leading to impairment of RNA processing [102,103,104]. Furthermore, many studies report widespread transcriptome changes in ALS carrying the *C9orf72* repeat expansion [102,105,106,107,108]. One report also highlighted the splicing factor hnRNP H as a major C9orf72 binding protein, which was linked to the formation of abnormal nucleic acid structures [109].

Another possible process associated with a gain of toxic function of the *C9orf72* repeat expansion is repeat-associated non-ATG dependent (RAN) translation [101], whereby expanded repeat sequences are translated in the absence of an ATG initiation codon. RAN translation has now been described for several non-coding repeat expansions, including *C9orf72*-ALS/FTD [110]. Recent studies have revealed that translation of the *C9orf72* repeat is initiated from a CUG codon upstream from the repeat sequence, which is induced in response to stress stimuli and depends on phosphorylation of the α-subunit of eukaryotic initiation factor-2 (eIF2α) [111,112,113]. In ALS/FTD, RAN translation produces dipeptide repeat proteins (DRPs), which result from translation on the both sense and antisense strands. This results in expression of five DPRs: poly GA, poly GR, poly PA, poly PR, and poly GP (which is produced on both sense and anti-sense strands). The biochemical properties of each DPR are quite distinct, and the arginine-containing peptides (poly GR and poly PR) appear to be the most toxic, at least in disease models [114,115]. Furthermore, these peptides display features associated with neurodegeneration, including liquid–liquid phase separation, perturbation of nucleocytoplasmic transport, and stress granule formation [116,117].

The cause of the selective neurodegeneration of motor neurons in ALS associated with *C9orf72* repeat expansions is unknown. However, there are several hypotheses, based on the unique characteristics of motor neurons. As explained above, neurons themselves are particularly susceptible to DNA damage. However, in addition, motor neurons are extremely large cells with high levels of cellular respiration, and thus they are especially prone to oxidative stress. This may render them particularly susceptible to DNA damage, even compared to other types of neurons. There are also other possibilities to explain the selective neurodegeneration of motor neurons in ALS. It has been shown that the excitabilities of corticospinal tract pathways are abnormally increased in ALS, especially in the early stages of disease [118], and an imbalance in excitatory to inhibitory synaptic input precedes motor neuron degeneration in animal models [119]. Interestingly, it has also been demonstrated that physiological neuronal activity causes DNA damage [120,121]. Whilst this may be effectively repaired in normal physiological conditions, the presence of the *C9orf72* repeat expansion may disrupt the natural cellular safeguarding mechanisms, thus contributing to neurodegeneration in ALS.

## 6. Abnormal Nucleotide Structures: R-Loops, G-Quadruplexes, and Hairpins

Nucleic acids are structurally polymorphic. Whilst the double-stranded, right-handed helix is the regular conformation employed by both DNA and RNA, non-canonical alternative structures, such as hairpins, branched junctions, and quadruplexes, also exist [122]. Normal cellular processes leading to transient separation of nucleic acid strands, such as DNA replication, recombination, repair and transcription, can lead to instability in their sequences. Not all non-canonical conformations are stable under physiological conditions, but increasing evidence links the formation of these structures with different biological functions and pathological conditions. Importantly, aberrant nucleic acid structures are increasingly acknowledged to be an important contributor to human disease [123,124]. They are also major sources of DNA damage and are thus recognised to be a serious threat to genomic integrity [125]. The ability of nucleic acids to form unusual secondary structures is also related to the instability of repeat sequences [126]. Below we discuss two important nucleic acid structures that are formed by the *C9orf72* repeat sequence.

G-quadruplex structures are formed in nucleic acids by G-rich sequences, which is not surprising because G-rich DNA is prone to forming stable secondary structures. G-quadruplexes contain G tetrad structures that are stacked on top of one another. G tetrads consist of four guanine bases Hoogsteen hydrogen-bonded to each other and a cation. They possess normal physiological roles, such as in immunoglobulin heavy chain switching [127], and they are often found at important positions in the genome, such as telomeres [128]. However, aberrant, detrimental roles have also been described in disease. G-quadruplexes are gaining increasing interest because of their involvement in signalling pathways that are relevant to cancer and neurodegeneration. In neurological disorders, G-quadruplexes have been implicated in pathogenesis through two main mechanisms. The first is by expansions of G-repeats, which lead to the formation of G-quadruplexes that induce toxicity, such as in *C9orf72*-ALS. The second mechanism is through mutations that affect the expression of G-quadruplex binding proteins, as in the fragile X mental retardation 1 (*FMR1*) gene and Fragile X syndrome [129].

R-loops are naturally occurring hybrids between DNA and RNA. They form when an RNA strand displaces a strand of the original DNA double helix, because the stability of the RNA–DNA interaction is greater than DNA–DNA interactions. Hence, the resulting R-loops can be extremely stable. The formation of R loops often occurs in G-rich sequences, again reflecting the propensity of single-stranded G-rich sequences to form stable secondary structures. R-loops occur normally during many cellular processes, including DNA replication, transcription (including reverse transcription), and telomere function, but in these situations, they normally form transiently and do not persist [130,131]. However, the persistence of R-loops can have deleterious effects, resulting in genome instability and DNA damage. This is mediated by at least two distinct mechanisms. Firstly, ssDNA that is exposed via an R-loop is chemically labile, and hence more prone to damage. Secondly, R-loops can block replication fork progression, leading to replication stress and error-prone repair mechanisms [130]. R loops can also lead to reduced protein expression by transcriptional stalling, or by negatively regulating RNA polymerases, thus inhibiting transcription [132]. Furthermore, R-loops can mediate other mechanisms of transcriptional repression, such as the methylation of histones [133,134]. R loops are therefore closely associated with RNA metabolism, which is implicated as a major pathogenic mechanism in ALS [135]. Therefore, it is not surprising that R-loops are linked to various diseases, including multiple cancers and neurodegenerative disorders. Normal cellular mechanisms exist to prevent the formation of R-loops, including senataxin [74]. Interestingly, *SETX*, the gene encoding senataxin, is mutated in juvenile ALS [58], as is ataxin with oculomotor apraxia type 2 (AOA2) [136].

## 7. The Chromosome 9 Open Reading Frame 72 Repeat Expansion Induces DNA Damage

The properties of the G-rich GGGGCC repeat render the *C9orf72* repeat expansion highly favorable for forming abnormal DNA structures, such as G-quadruplexes and R-loops [137,138,139]. Circular dichroism (CD) and nuclear magnetic resonance spectroscopy (NMR) studies [140,141] have revealed that the *C9orf72* repeat expansion forms a heterogenous mixture of G quadruplex conformations, involving both parallel and anti-parallel G structures. Importantly, this interferes with the function of RNA polymerase at repeat sites, leading to abortive transcripts and less full-length transcripts [140]. Treatment with RNase A and RNase H to remove RNA alters the mobility of in vitro transcription products, also providing evidence for the presence of R-loops [140]. In addition, more R-loops were detected by immunohistochemistry in spinal cord motor neurons from C9orf72 ALS patients, compared to controls [46]. Similarly, expression of DPRs in cell culture resulted in the production of more R-loops by immunocytochemistry, which could be reduced by expression of senataxin [46]. R-loops are often found at cytosine–phosphate–guanine (CpG) islands and are proposed to suppress DNA methylation [142]. Interestingly, there are two CpG islands flanking the *C9orf72* repeat expansion that are differentially methylated [143].

The formation of G-quadruplexes and R-loops by the *C9orf72* repeat expansion implies that these structures could damage DNA. Consistent with this notion, elevated levels of DNA damage markers γH2AX, ATR, GADD45, and p53 were present in motor neurons differentiated from iPSC lines from *C9orf72* ALS patients in response to oxidative stress, which could be reduced by pharmacological or genetic reduction of oxidative stress [144]. Similarly, we demonstrated that markers of the DDR, including γH2AX, phosphorylated-ATM, cleaved PARP-1, and 53-BP1, were up-regulated in *C9orf72* ALS patient spinal cord motor neurons [47]. This was confirmed using constructs expressing poly (GR)_100_ and poly (PR)_100_, but not the native GGGGCC RNA, revealing that DNA damage is activated by the DPRs produced by RAN translation of the *C9orf72* repeat expansion in ALS. A subsequent study also found that the DPRs induce DNA damage, and that in addition, the *C9orf72* RNA is capable of inducing damage [30]. Expression of the C9orf72 DPRs resulted in suppression of the recruitment of 53BP1 to DSBs. This led to defective ATM signalling and hence DNA repair, which appeared to be driven by the accumulation of p62, and subsequently, defective H2A ubiquitylation. However, a second mechanism was also implicated; the persistent accumulation of R-loops resulting in the formation of DSBs, increased heterochromatin, and splicing defects [46].

## 8. The Nucleolus and the Chromosome 9 Open Reading Frame 72 Repeat Expansion

The main function of the nucleolus is the rapid production of ribosomal subunits, a process that must be highly regulated to achieve proper cellular proliferation and cell growth [145]. This involves three main events: pre-rRNA transcription, processing, and ribosomal RNP assembly. These functions are concentrated in three distinct sub-nucleolar compartments, the fibrillar center (FC), the dense fibrillar component (DFC), and the granular component (GC). The varied effects on ribosome subunit production and cell growth induced by cellular stress are often accompanied by dramatic changes in the organization and composition of the nucleolus, and the nucleolus is recognised to be a central hub in cellular stress responses. During DNA damage, the nucleolus segregates, resulting in condensation and separation of FC and GC, as well as the formation of ‘‘nucleolar caps’’ around the nucleolar remnant (also called central body) [146].

Dysfunction in the nucleolus is now implicated as an important mechanism related to toxicity of the *C9orf72* repeat expansion. The *C9orf72* repeat RNA binds nucleolar proteins in vitro [104,140]. Over-expression of poly GR or poly PR repeats in cell culture leads to their localisation in the nucleolus, resulting in abnormal nucleoli, altered ribosomal RNA processing, nucleolar stress, and cell death [140,147,148]. Additionally, in yeast several nucleolar proteins modify poly PR toxicity [116]. Furthermore, nucleolin, an important nucleolar protein involved in the synthesis and maturation of ribosomes, binds specifically to G-quadruplexes formed by the *C9orf72* repeat expansion [140]. Whilst the C9orf72 DPRs do not localise to the nucleolus in *C9orf72*-ALS brains, their neuronal nucleoli display abnormalities [149]. Disrupted nucleocytoplasmic transport is emerging as a central pathogenic mechanism in ALS that is also closely associated with nucleolar stress. The C9orf72 DPRs inhibit nuclear import and export, and enhancement of nuclear import or suppression of nuclear export, suppressed neurodegeneration in yeast and Drosophila [116,150].

Nucleophosmin (NPM1, also known as B23) is a nucleolar-localised DDR protein that regulates nucleolar function and contributes to genomic integrity and stability [151,152]. During DNA damage, NPM1 localises to DSBs, where it mediates the stability, activity, and accumulation of proteins involved in base excision DNA repair (BER) [153]. BER corrects small base lesions, typically resulting from deamination, oxidation, or methylation, which do not significantly distort the DNA helix structure. NPM1 also interacts with APE1, which is central to BER [154]. The NPM1-APE1 interaction regulates multiple cellular functions, including genomic stability and ribosome biogenesis [155]. APE1 is also a growth factor, which is protective against apoptosis induced by DNA damage [156]. Under normal conditions, NPM1 enhances the activity of APE1, thus enhancing BER [157,158,159]. However, during nucleolar stress, NPM1 inhibits the activity of APE1, leading to impairment of BER [157,159,160]. Up-regulation of APE1 was previously reported in sporadic ALS patients [66], and missense mutations in APE1 were found in sporadic and familial SOD1-ALS patients [67]. NPM1 co-localises with both poly GR and poly PR [148]. In *C9orf72* ALS patients, we showed that the interaction between NPM1 and APE1 was enhanced compared to control subjects, which may impair the function of both, and in turn disturb RNA processing [47].

**Figure 1:**
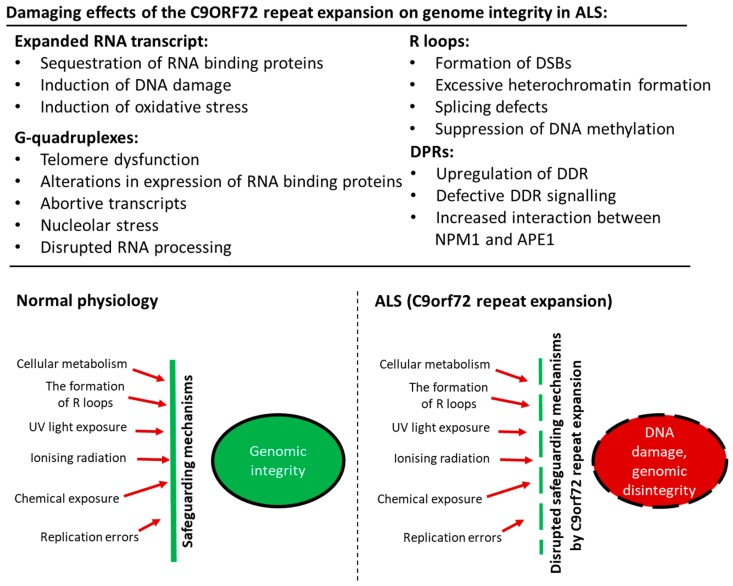
Scheme illustrating mechanisms by which genomic integrity is disrupted by the *C9orf72* repeat expansion in ALS. Cells are exposed to exogenous and endogenous sources of DNA damage, such as normal cellular metabolism, the formation of R-loops, UV light exposure, ionising radiation, chemical exposure, and replication errors. In normal physiological conditions, the integrity of the genome is preserved by safeguarding mechanisms: the DDR and the nucleolus. However, in ALS, transcription of the *C9orf72* repeat expansion leads to the production of expanded RNA transcripts. Furthermore, DPRs and abnormal DNA structures, such as R-loops, hairpins, and G-quadruplexes, are formed. These conformations compromise the normal cellular protective mechanisms, leading to persistent DNA damage and loss of genomic integrity.

Figure 1 summarises possible mechanisms by which genomic integrity is disrupted by the *C9orf72* repeat expansion in ALS, as discussed in the sections above.

## 9. Novel Therapeutic Strategies for Amyotrophic Lateral Sclerosis Based on the Inhibition of DNA Damage and Abnormal DNA Structures.

The increasing evidence that both the repeat RNA and DPRs contribute to toxicity implies that therapeutic strategies based on targeting both factors may be effective in ALS. In addition, there is evidence that the *C9orf72* repeat expansion is upstream of the TDP-43 pathology which is present in almost all ALS cases [161,162], further implying that targeting this region could be an effective strategy in ALS. Abnormal nucleic acid structures, such as G-quadruplexes and R-loops, are increasingly recognised as promising drug targets, and several small molecules are being developed to target these sites. 

There are several lines of evidence implying that the targeting the G-quadruplexes formed by the *C9orf72* repeat expansion could be an effective therapeutic strategy for *C9orf72*-ALS and FTD. Targeting the hairpin conformation of the *C9orf72* repeat expansion with small chemical lead compounds was protective against the formation of RNA foci and RAN translation [163]. Recently, three small molecules were isolated from chemical libraries that bind and stabilise the *C9orf72* G-quadruplex structure [164]. Moreover, these compounds reduced the formation of both *C9orf72* RNA foci and DPRs in iPSC-derived motor neurons, and they improved survival in fly models expressing poly GR [164]. A porphyrin compound, TMPyP4, also stabilized *C9orf72* G-quadruplexes, reduced the affinity of RanGAP1, a key regulator of nucleocytoplasmic transport, and suppressed nuclear import deficits in fly models [150]. 

Recent studies indicating that DNA repair pathways are dysregulated by the *C9orf72* repeat expansion implies that modulation of the DDR or enhancement of DNA repair processing are also promising therapeutic strategies for ALS. Indeed, there are already many compounds in development that modulate the DDR for cancer therapy. Hence, similar approaches could be examined for ALS and other neurodegenerative disorders. An important tumour suppressor, p53, protects the genome by regulating a variety of DDR mechanisms and controls the induction of apoptosis in genomically compromised cells. Not surprisingly, a broad range of approaches for modulating or inhibiting p53 activity are already underway in studies of cancer therapy [165,166,167]. Interestingly, partial inhibition of the p53 pathway partially suppressed poly GR_80_ induced toxicity in iPSC-derived patient motor neurons [144]. Another approach is based on the impairment of ATM activation and the suppression of 53BP1 recruitment to DSBs [46] by the *C9orf72* repeat expansion. This implies that inhibition of negative regulators of DNA repair, such as the PI3K/AKT mammalian target of rapamycin (mTOR) pathway, which negatively controls ATM [168], may be beneficial for ALS. Consistent with this notion, we also demonstrated the down-regulation of PI3K and p-eIF4G in *C9orf72* patient tissues compared to controls [47], in concordance with previous studies demonstrating dysregulation of the AKT/PI3K pathway in ALS motor neurons [169,170,171]. Alternatively, D52 is another recently recognised negative regulator of ATM signalling [172]. Similarly, nucleolar stress also triggers down-regulation of the mTOR pathway [173], and the evidence that the *C9orf72* repeat expansion induces nucleolar stress implies that targeting this mechanism could be an important therapeutic strategy in ALS. We also previously showed that overexpression of NPM1 inhibited apoptosis in neuronal cells expressing poly (PR)_100_ or poly (GR)_100_, suggesting that depletion of NPM1 is linked to cell death in ALS [47]. These findings suggest that inhibition of nucleolar stress should be investigated in more detail in relation to *C9orf72*-ALS.

Ultimately however, a potentially more effective way to reduce toxicity of the *C9orf72* repeat expansion is by inhibiting its production in the first place. Antisense oligonucleotides (ASOs) are short, single-stranded oligonucleotides that can be designed to hybridize to specific RNAs and thus modulate gene expression [174]. Importantly, ASOs targeting the *C9orf72* repeat expansion are currently showing promise for ALS. ASO treatment targeting poly GP reduced both repeat-containing RNA foci and poly GP concentrations in *C9orf72* ALS iPSC-derived neurons, although poly GP is particularly stable and required 10 days of ASO treatment to be significantly reduced [175]. In ASO-treated mice, concentrations of *C9orf72* repeat-containing RNA were reduced approximately 50%, without affecting endogenous *C9orf72* mRNA. Similarly, poly GP concentrations in cerebrospinal fluid (CSF) and the brain, as well as RNA foci and poly GP-containing inclusions, were reduced significantly in the motor cortex of the mouse model of *C9orf72* ALS [175]. Furthermore, ASOs targeting *C9orf72* RNA prevented nuclear import impairment by the *C9orf72* repeat expansion in fly models, as well as in *C9orf72* iPSC-derived neurons, and suppressed neurodegeneration [150]. In addition, ASOs selectively reduced the accumulation of *C9orf72* GGGGCC sense strand-containing RNA foci, without significantly affecting the level of RNAs encoding C9orf72 itself. Similarly, ASOs targeting the *C9orf72* transcript suppressed GGGGCC repeat-containing RNA foci formation, and reversed membrane excitability defects in *C9orf72*-ALS motor neurons differentiated from iPSCs [106]. Hence these studies reveal that ASOs are capable of reducing pathogenic, expansion-containing RNAs without inducing C9orf72 protein loss [176].

## 10. Conclusions

**Figure 2:**
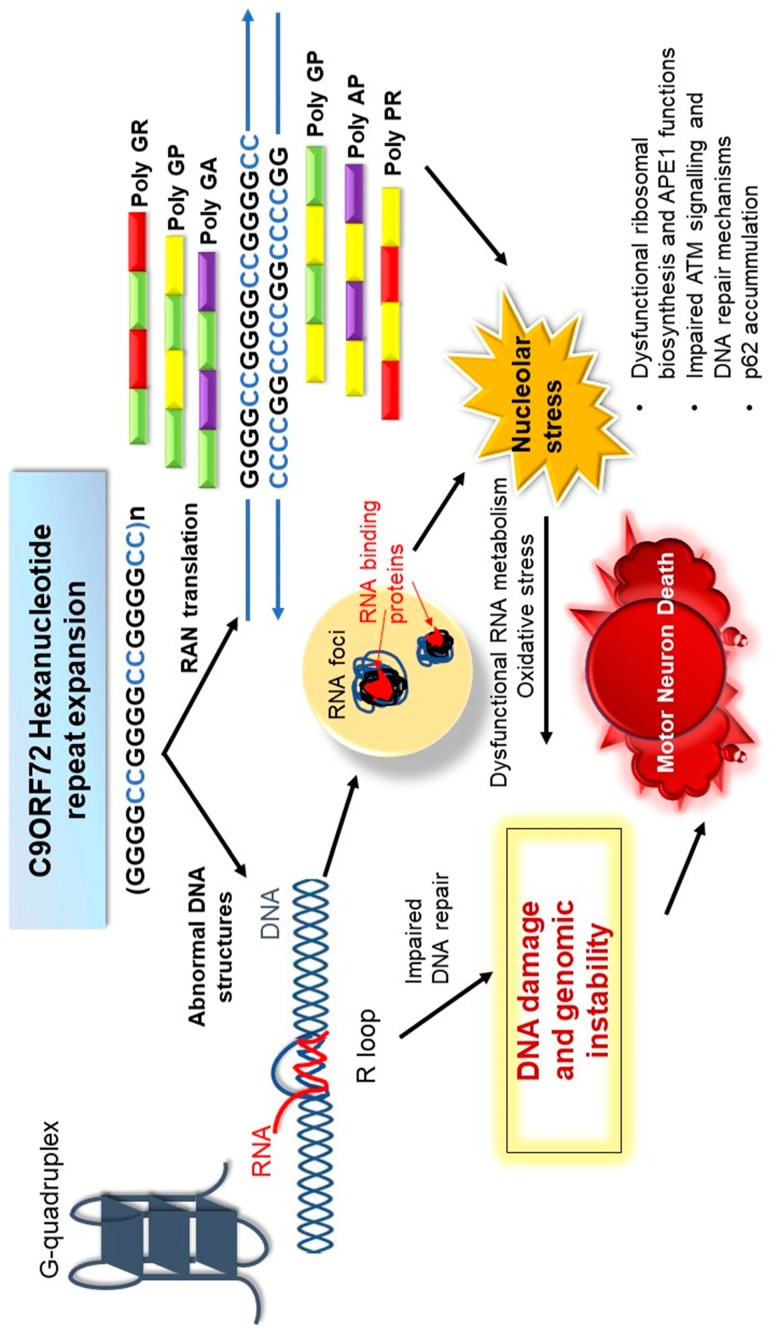
Mechanisms of amyotrophic lateral sclerosis (ALS) pathogenesis induced by the chromosome 9 open reading frame 72 (*C9orf72*) repeat expansion. The *C9orf72* repeat expansion forms abnormal nucleic acid structures, including R-loops, which perturb DNA repair processes involving ATM, and probably other mechanisms. The expanded RNA forms foci and sequesters RNA binding proteins, leading to dysfunction in RNA processing. The C9orf72 DPRs, produced by RAN translation, also accumulate in the nucleolus, leading to perturbations in nucleolar function, including DNA repair processes, ribosomal biogenesis, and APE-dependent mechanisms (including oxidative stress). They also impair DNA repair by p62-dependent mechanisms. Together, these events result in the accumulation of DNA damage, genome instability, and motor neuron death.

There is now convincing evidence that diseases resulting from the expansion of abnormal repeat sequences are nucleic acid diseases, and in *C9orf72*-ALS, DNA damage and loss of genome integrity are implicated in pathophysiology. In Figure 2, we illustrate the possible mechanisms by which the *C9orf72* mutation induces toxicity and genomic instability in ALS. The production of aberrant, toxic nucleic acid conformations formed by the *C9orf72* hexanucleotide (GGGGCC) repeat expansion (particularly R-loops) induces DNA damage. This results from impairment of DDR signalling and dysfunction in RNA processing, which compromises genetic integrity and triggers neurodegeneration. Furthermore, the DPRs produced by RAN translation of the *C9orf72* repeat expansion, particularly the arginine-containing peptides poly GR and poly PR, are also capable of triggering DNA damage by inducing stress in the nucleolus. They also perturb DNA repair by p62 accumulation and impairing NPM1–APE1 functions. In addition, oxidative stress involving APE1 also further compounds the damage. Hence, expression of the *C9orf72* repeat expansion results in a “double whammy” for the cell: both RNA- and DPR-driven mechanisms conspire to produce extensive DNA damage and genomic instability, leading to motor neuron death. Further studies are now warranted to determine exactly how DNA repair mechanisms are compromised in ALS, the extent to which the DDR is induced, and which specific pathways are compromised. Modulation of the DDR, enhancement of DNA repair processes, and the targeting of G-quadruplexes and R-loops, may therefore be novel neuroprotective strategies for ALS that should be harnessed in future studies.
